# Non-suicidal self-injury and suicidal ideation among adolescents: the chain-mediating role of rumination and decentering

**DOI:** 10.3389/fpsyt.2023.1179518

**Published:** 2023-09-15

**Authors:** Yinwei Zheng, Jing Wang, Qin Jiang, Meiling Liao, Fajie Huang

**Affiliations:** School of Health, Fujian Medical University, Fuzhou, China

**Keywords:** adolescent, non-suicidal self-injury, suicidal ideation, rumination, decentering

## Abstract

**Objective:**

To explore the relationship between non-suicidal self-injury and suicidal ideation in adolescents and examine the roles of rumination and decentering in that relationship.

**Method:**

By means of a questionnaire, 175 adolescent patients in a psychiatric hospital in Fujian Province were given the Functional Assessment of Self-Mutilation: Chinese Version, Positive and Negative Suicide Ideation, Ruminative Response Scale: Chinese Version, and Experiences Questionnaire: Decentering Scale.

**Results:**

(1) Adolescent non-suicidal self-injury was significantly positively related to suicidal ideation and rumination and significantly negatively related to decentering. Suicidal ideation was significantly positively related to rumination and significantly negatively related to decentering. Rumination was significantly negatively related to decentering. (2) Rumination and decentering played a complete chain-mediating role between non-suicidal self-injury and suicidal ideation. Non-suicidal self-injury was found to indirectly affect suicidal ideation along three pathways: the independent mediating role of rumination (the mediating effect accounted for 40.166%), independent mediating role of decentering (the mediating effect accounted for 41.274%), and chain-mediating role of rumination and decentering (the mediating effect accounted for 14.958%).

**Conclusion:**

Adolescent non-suicidal self-injury can indirectly affect suicidal ideation through rumination and decentering. In the future, mindfulness and other methods should be used to improve individuals’ levels of decentering and cultivate emotional regulation abilities, so as to reduce the incidence of non-suicidal self-injury and suicide in adolescents.

## 1. Introduction

Non-suicidal self-injury (NSSI) refers to self-harm behaviors that can lead to physical injury but are exhibited in the absence of an intent to die; such behaviors are not accepted by society or in culture (1). Common forms of NSSI include skin cutting, tearing, and impacting, and usually share three characteristics: repeatability, intentionality, and self-concealment (2). At present, NSSI is listed as an independent diagnosis in the third part of the fifth edition of the Diagnostic and Statistical Manual of Mental Disorders (3). Previous studies have shown that the detection rate of NSSI in Chinese adolescents is generally higher than what is seen in Western countries (4) and is showing a gradually increasing trend (5). NSSI not only causes physical damage to adolescents, but also greatly harms their psychology, including increasing the likelihood of certain diseases, various psychological and behavioral disorders, and even suicidal behavior (6, 7). Among them, the risk of suicide in patients engaging in NSSI increases by more than seven times (8). Therefore, NSSI in adolescents has become an important mental health and scientific research issue.

Suicidal ideation refers to “plans and desires to commit suicide that have not yet been attempted” (9). Although suicidal ideation is only the initial stage of suicidal behavior, it is a strong risk factor for suicide (10). Investigating the relationship between NSSI and suicidal ideation will be helpful for early intervention with people at high risk of suicide, better ensuring the health and quality of life of patients. However, the interpretation of suicidal ideation as the initial stage of suicidal behavior, in association with different forms of adolescent NSSI, is not universal. Some studies have suggested that adolescent NSSI can trigger a variety physiological effects and negative emotions or thoughts, leading to a diversion of attention; it also may have a positive effect on improving interpersonal conflict and obtaining a high level of social support, thus reducing the possibility of individuals engaging in suicidal behavior (11, 12). Therefore, NSSI may have the function of anti-suicide in adolescents (13). Other studies have suggested that there is a significant association between NSSI and suicidal ideation, which often occur simultaneously, and most suicidal psychological actors have engaged in NSSI (14). A past history of NSSI has been identified as a predictor of suicidal ideation (15). Moreover, studies have used the two self-injury purposes of “making me cry” and “hoping someone notices what’s wrong” as indicators of suicidal ideation, and directly tested whether NSSI may be an important predictor of suicidal ideation (16). Based on this, the present study proposes Hypothesis 1: NSSI can positively predict suicidal ideation.

Rumination is a phenomenon in which individuals automatically and repeatedly focus on their thoughts after experiencing a negative event (17). Numerous studies have shown that rumination is a powerful influential factor in NSSI and suicidal behavior (18–20). However, few studies have directly examined how rumination operates between the two. First, it has been found that individuals with a past history of NSSI tend to have a higher tendency to ruminate (21). Recent NSSI can significantly positively predict rumination (22). Secondly, the emotional cascade theory holds that an individual’s rumination will magnify negative emotions and their effects, even if they serve as only a minor emotional stimulus. Rumination often leads people to constantly focus on re-experiencing the effects of negative emotions, which causes them fall into a vicious cycle and become more painful. Therefore, in order to avoid or alleviate negative emotions, they are more likely to resort to self-harm (23), resulting in higher levels of suicidal ideation (24). A substantial number of studies have shown that there is a significant positive correlation between rumination and suicidal ideation (25, 26), and rumination can significantly positively predict individual suicidal ideation (25, 27). Based on the above analysis, this study speculated that individuals with NSSI would have a greater tendency to ruminate, which could lead to more negative emotions and make suicidal ideation or even suicidal behavior more likely. Therefore, this study proposes Hypothesis 2: Rumination plays a mediating role in the relationship between NSSI and suicidal ideation.

Based on the above literature review, the diathesis-stress model (DSM) was selected to provide the theoretical basis for this study, which examines the relationship between NSSI and suicidal ideation in adolescents. The DSM holds that suicidal ideation originates from the interaction between biological and behavioral tendencies, combined with self-destructive impulses and stressors or triggers such as recent life events (28). Under the framework of the DSM, this study argues first that under the influence of external stress events, individuals tend to adopt NSSI as a negative problem-solving approach. However, this cannot release their stress emotions and instead will cause a greater negative emotional state. Second, an individual’s NSSI behavior will automatically enhance their tendency toward ruminative thinking, which will not only force them to continue to pay attention to and re-experience negative emotions, but also magnify those negative emotions and their impact. Therefore, under the influence of external stress events and the sequential negative processing of internal ruminative thinking, individuals will experience greater psychological pressure that will lead to greater suicidal ideation.

However, the DSM does not explain why rumination affects suicidal ideation. There has been a lack of discussion on this issue, but the present study’s introduction of the concept of decentering may help to further explain the issue. Decentering, also known as self-alienation or psychological alienation, refers to the ability to distance oneself from an internal experience and recognize the gap between objective reality and the subjective reality constructed by the individual (29). It is worth noting that on the one hand, studies have shown that the tendency toward rumination in NSSI individuals can cause them to be more emotionally distressed, showing typical dissociative symptoms (30) such as the feelings of emptiness, numbness, and self-alienation that NSSI individuals often report (31). The essence of dissociative symptoms is confusion in terms of self-identity, which is manifested by the world feeing unreal and a reduction of the consciousness ability of the self to operate in the objective world. Thus, the dissociative symptoms experienced by NSSI individuals may be the result of the pain caused by rumination. As such, this study suggests that rumination in NSSI individuals will negatively predict decentering. On the other hand, in terms of the pain caused by rumination, NSSI individuals often experience suicidal ideation and may even commit suicide to resolve their feelings of emptiness, numbness, and self-alienation and dissociation. Moreover, studies have shown that mindfulness-based cognitive therapy with decentering, attentional control, and mindfulness awareness as operating mechanisms (32) may reduce rumination in an individual during treatment (33–35), increase the level of decentering, and ultimately lead to a decrease in suicidal ideation (36–39). Moreover, Lo et al. (40) found that decentering could weaken the effect of a ruminative self-focus in depressed individuals, thereby reducing automatic negative thoughts and stopping the vicious cycle of rumination. In other words, decentering can further reduce the negative effects caused by rumination (including suicidal ideation), meaning that decentering will also negatively predict suicidal ideation. Thus, NSSI individuals may show more dissociative symptoms after experiencing the pain of rumination, and the level of decentering will decrease. In order to be rid of the emptiness, numbness, and self-alienation of the dissociative state, NSSI individuals may eventually experience more suicidal ideation or even commit suicide. Based on this, the present study proposes Hypothesis 3: Decentering plays a mediating role in the relationship between rumination and suicidal ideation.

In addition, there is substantial evidence that decentering is an effective regulatory strategy for alleviating the harmful effects of rumination. For example, Kaiser et al. (41) found that rumination and decentering were negatively correlated and rumination might serve to impede the ability to clear irrelevant information from the working memory when under negative emotional conditions. Eftekhari et al. (42) demonstrated that decentering not only reduces problematic symptoms such as rumination and anxiety, but also increases the approach motivation of depressed individuals. Based on the above literature analysis, adolescents with NSSI tend to have more negative emotions (43). However, NSSI causes individuals to have a higher tendency to ruminate, which also leads to a lower level of decentering, making it difficult to alleviate negative emotions and ultimately leading to an increase in the level of suicidal ideation. Based on this assumption, this study proposes Hypothesis 4: Rumination and decentering play a chain mediation role in the relationship between NSSI and suicidal ideation.

It should be noted that Hypothesis 4 contains a relationship that has not been analyzed in detail in previous research: the effect of NSSI on decentering. Individuals with NSSI usually have dissociative symptoms and feel the absence caused by feelings of emptiness, numbness, and self-alienation (31). Some studies have found a significant positive correlation between NSSI and dissociative symptoms (30). Dissociation can make it difficult for NSSI individuals to construct a complete reality (44), and the level of decentering seen in NSSI individuals decreases throughout this process. According to predictive processing (PP) theory, in an uncertain world, individuals need to construct themselves through the process of action, give specific meaning to their actions, and construct a cognitive model about the self and world that emphasizes the unity of cognition and action in subsequent life (45, 46). According to PP theory, because an individual’s NSSI behavior itself is a biased response, the negative self-perception given by that response is automatically activated, leading to the improvement of a centralized level of NSSI. Accordingly, this study speculates that NSSI negatively predicts decentering. As mentioned above, decentering negatively predicts suicidal ideation; that is, NSSI individuals may experience more suicidal ideation or even commit suicide in order to rid themselves of the sense of emptiness, numbness, and self-alienation that accompanies dissociation. Based on the above analysis, this study adds Hypothesis 5: decentering plays a mediating role between NSSI and suicidal ideation.

**Figure 1:**
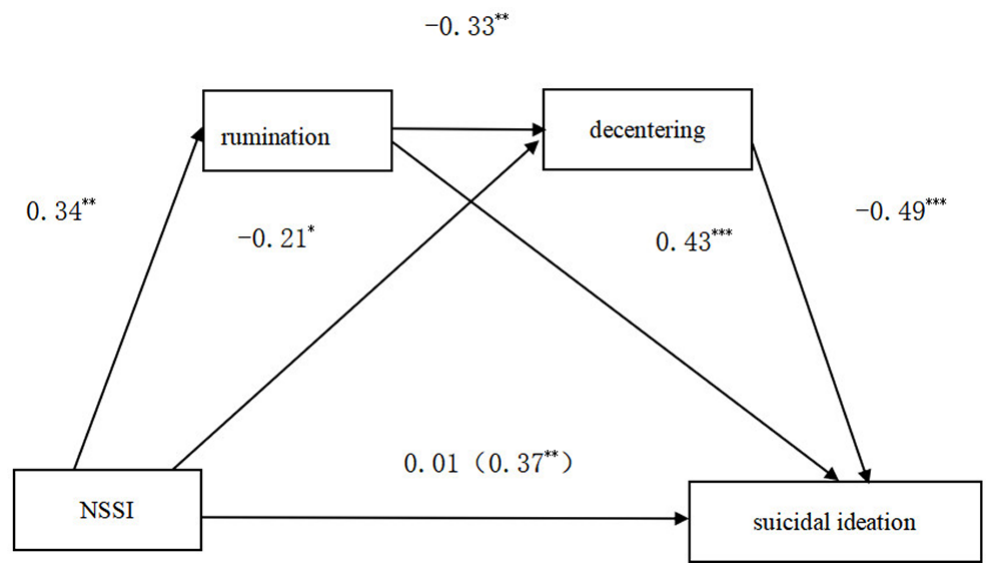
Theoretical construction model of rumination and decentering.

Based on a review of previous research results, as shown in Figure 1, this study proposes five hypotheses regarding the relationship between adolescent NSSI and suicidal ideation: (1) NSSI can positively predict suicidal ideation, (2) rumination plays a mediating role in the relationship between NSSI and suicidal ideation, (3) decentering plays a mediating role in the relationship between rumination and suicidal ideation, (4) rumination and decentering play a chain mediating role in the relationship between NSSI and suicidal ideation, and (5) decentering plays a mediating role between NSSI and suicidal ideation. We hope to provide references for effective interventions in NSSI behavior by exploring the relationships and underlying mechanisms operating among NSSI, suicidal ideation, rumination, and decentering.

## 2. Method

### 2.1. Participants

From September 2021 to January 2022, a convenience sampling method was used to distribute a questionnaire survey in a psychiatric hospital in the Fujian Province of China. Patients were enrolled in the study if they met the following initial inclusion criteria: (1) met the diagnostic criteria of the ICD-10, (2) had reading and expression abilities, and (3) consented to participate in the survey. The exclusion criteria included an: (1) inability to cooperate with the survey, (2) inability to communicate normally with the investigators, and (3) presence of severe psychotic symptoms. All patients signed an informed consent form and minor patients obtained informed consent from their parents. A total of 192 questionnaires were distributed and 175 valid questionnaires received, for a recovery rate of 91.146%. Among the valid respondents, 38.286% were males (*N* = 67), 61.732% were females (*N* = 108), 26.857% had no siblings (*N* = 47), 73.143% had siblings (*N* = 128), 52.571% were urban patients (*N* = 92), 30.857% resided in townships (*N* = 54), and 16.572% lived in rural areas (*N* = 29). The ages of the participants ranged between 12 and 25 years (*M* = 18.610, *SD* = 3.552).

### 2.2. Measures

#### 2.2.1. Chinese functional assessment of self-mutilation

The English version of the Chinese Functional Assessment of Self-Mutilation(C-FASM) scale was compiled by Lloyd. In the present study, the Chinese version was revised by Wenshu et al. and used to assess the frequency, motivation, and types of NSSI behaviors occurring in the previous 12 months (47). All 11 types of NSSI behaviors were evaluated using this scale. Participants were divided into NSSI and non-NSSI groups, according to whether or not they had engaged in self-injury in the past year. Participants were then further divided into mild, moderate, and severe groups, according to the level of self-injury. In addition to the method and frequency of NSSI, respondents were asked to indicate their NSSI motivation via 22 items; each was answered on a four-point scale ranging from 0 (never) to 3 (often). The Cronbach’s α coefficient was 0.923 for this study.

#### 2.2.2. Positive and negative suicidal ideation

The adolescent suicidal ideation scale was revised by Xuezhi et al. It was used in the present research to evaluate the suicidal ideation of respondents in the previous 2 weeks (48). The scale was divided into two dimensions: positive and negative suicidal ideation. It included 14 items answered on a five-point scale ranging from 1 (never) to 5 (always). The positive suicidal ideation dimension was scored in reverse; otherwise, higher scores represented higher levels of suicidal ideation. The Cronbach’s α coefficient was 0.947 for this study.

#### 2.2.3. Ruminative response scale

The English version was compiled by Nolen-Hoeksema. The present study used the Chinese version, which was revised by Xiu to evaluate rumination tendencies in Chinese samples (49). The scale was divided into three dimensions: symptom rumination, brooding, and reflective pondering. It included 22 items answered on a four-point scale ranging from 1 (never) to 4 (always). Higher scores indicated greater rumination tendencies. The Cronbach’s α coefficient was 0.918 for this study.

#### 2.2.4. Experiences questionnaire—decentering

The English version of this measure was compiled by Fresco et al. (50). There were 11 items, each rated on a five-point Likert scale ranging from 1 (very non-conforming) to 5 (very compliant). Higher scores indicated a higher level of decentering. The Cronbach’s α coefficient was 0.805 for this study.

### 2.3. Statistical analysis

The data were processed using SPSS 26.0 to calculate the descriptive statistics, an independent samples *t*-test, one-way ANOVA, Harman’s single-factor testing, and Pearson correlation coefficients. The models were tested in PROCESS for chain mediation.

## 3. Results

### 3.1. Common method deviation

Because multiple variables in this study were provided by the same people completing the questionnaire, there was a possibility that our data may have suffered from the common method bias effect.

Previous research has found that Harman’s single-factor test can be useful in evaluating the influence of common method bias (51). The measurement items for all variables used in this study were included in a factor analysis, which showed that 20 factors were extracted. The variance for the first factor was only 21.422%, less than the critical criterion of 40%. This indicated that common method bias was not a serious issue in the present research.

### 3.2. Preliminary analyses

In order to test the correlations among NSSI, suicidal ideation, rumination, and decentering, a Pearson correlation analysis was carried out. The results showed that there were no significant relationships between gender and each variable. NSSI was positively associated with suicidal ideation (*r*_1_ = 0.279, *p*_1_ < 0.01) and rumination (*r*_2_ = 0.243, *p*_2_ < 0.05) and negatively associated with decentering (*r*_3_ = −0.262, *p*_3_ < 0.01). Moreover, decentering was negatively associated with suicidal ideation (*r*_4_ = −0.638, *p*_4_ < 0.01) and decentering was negatively associated with rumination (*r*_5_ = −0.299, *p*_5_ < 0.01). Suicidal ideation was positively associated with rumination (*r*_6_ = 0.587, *p*_6_ < 0.01). Age was significantly correlated with the other variables. Therefore, it was treated as a control variable in the subsequent analysis. The results are shown in Table 1.

**Table 1:** Means, *SD*, and correlation matrix (*N* = 179).

Variable	*M*	*SD*	1	2	3	4	5	6
1. Gender			1					
2. Age	18.61	3.55	−0.043	1				
3. NSSI	30.41	16.10	−0.026	−0.120	1			
4. Suicidal ideation	41.05	13.92	0.064	−0.180^*^	−0.279^**^	1		
5. Rumination	57.59	14.59	0.265	0.076	0.243^*^	0.587^***^	1	
6. Decentering	32.76	7.74	−0.073	0.229^**^	−0.262^**^	−0.638^***^	−0.299^***^	1

^*^p < 0.05; ^**^p < 0.01; ^***^p < 0.001.

The C-FASM results showed that 111 participants (63.428%) reported a recent history of NSSI (i.e., within the past year). Among them, 31.531% reported mild NSSI and 68.469% indicated moderate/severe NSSI. The majority (72.072%) engaging in NSSI were between the ages of 12 and 18 years. It is worth noting that 11.711% of the NSSI occurred in individuals less than 12 years old and 16.217% occurred in those older than 18 years. The average age range for self-injury was between 14 and 21 years (17.810 ± 3.556).

### 3.3. Chain-mediated effect test

In this study, age was used as the control variable. The Bootstrap method provided by the SPSS macro program Process v3.3 was used, the number of repeated samplings was set to 5,000, and the default 95% confidence interval was employed to test the mediating effects of rumination and decentering operating between NSSI and suicidal ideation. Because the relationship between NSSI and suicidal ideation in the mild NSSI group was weak, it may not reflect the actual mechanism of the relationship between them. Therefore, the subjects were divided into three types for data analysis: the total NSSI group (without distinguishing the degree of NSSI), mild NSSI, and moderate/severe NSSI (47, 52). This study then continued the previous data analysis method by dividing the samples into three groups and testing the chain mediating effects of rumination and decentering.

First, in the total NSSI model, the indirect effect of NSSI on suicidal ideation through rumination was 0.077, but the bootstrap 95% confidence interval for this pathway contained 0 and did not reach a significant level. In the mild NSSI model, the bootstrap 95% confidence intervals for all three paths contained 0 and did not reach a significant level. That is to say, both the total NSSI and mild NSSI models were not valid.

Second, in the moderate/severe NSSI model, NSSI significantly positively predicted suicidal ideation (*β* = 0.365, *p* < 0.05). However, after incorporating rumination and decentering into the regression equation, the predictive effect of NNSI on suicidal ideation became insignificant (*β* = 0.184, *p* > 0.05). The specific relationships among the variables in the model are shown in Table 2 and manifested as follows. NSSI positively predicted rumination (*β* = 0.331, *p* < 0.01) and negatively predicted decentering (*β* = −0.304, *p* < 0.01); rumination negatively predicted decentering (*β* = −0.332, *p* < 0.15) and positively predicted suicidal ideation (*β* = 0.438, *p* < 0.01); and decentering negatively predicted suicidal ideation (*β* = −0.488, *p* < 0.001).

**Table 2:** Regression analysis of the moderate/severe NSSI model.

Outcome variable	Predictor variable	*R* ^2^	*β*	*t*
Suicidal ideation	Age	0.131	0.331	0.301
	NSSI		0.365	3.32^**^
Rumination	Age	0.125	0.176	1.595
	NSSI		0.331	2.993^**^
Decentering	Age	0.292	0.169	1.66
	NSSI		−0.304	−2.873^**^
	Rumination		−0.332	−3.134^**^
Suicidal ideation	Age	0.615	0.01	0.128
	NSSI		0.184	0.221
	Rumination		0.438	−5.22^**^
	Decentering		−0.488	−5.571^**^

The data were all standardized and input into the model for processing. ^**^p < 0.01.

**Figure 2:**
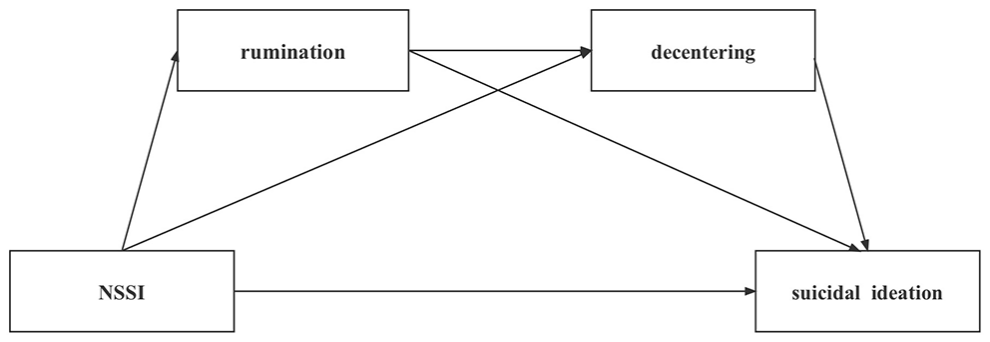
Chain-mediated model of rumination and decentering. *p<0.05; **p<0.01; ***p<0.001.

Third, the testing of the moderate/severe NSSI model showed that the chain mediating model held. Specifically, as shown in Table 3, the analysis results indicated that NSSI had no significant direct predictive effect on suicidal ideation. Rumination and decentering played mediating roles between NSSI and suicidal ideation. The mediating effect value was 0.347, accounting for 96.12% of the total effect. Further analysis showed that the mediating effect was composed of indirect effects generated along three paths. (1) For NSSI-rumination-suicidal ideation, the indirect effect was 0.145, accounting for 40.166% of the total effect. (2) For NSSI-decentering-suicidal ideation, the indirect effect was 0.149, accounting for 41.274% of the total effect. (3) For NSSI-rumination-decentering-suicidal ideation, the indirect effect was 0.054, accounting for 14.958% of the total effect. The bootstrap 95% confidence intervals for the three pathways did not contain 0, indicating that the mediating effects of these three pathways reached a significant level (Figure 2).

**Table 3:** Mediating effect analysis of the moderate/severe NSSI model.

Mediating path	Effect	BootSE	BootLLCL	BootULCL	Effect size
Indirect effect 1	0.145	0.054	0.492	0.258	40.17%
Indirect effect 2	0.149	0.046	0.059	0.238	41.27%
Indirect effect 3	0.054	0.028	0.01	0.12	14.96%
Total indirect effect	0.347	0.087	0.171	0.514	96.40%

## 4. Discussion

### 4.1. The detection rate of NSSI among adolescents

Adolescence is a period that sees a high incidence of NSSI, and the detection rate of self-injury among Chinese youth shares this characteristic. At the same time, adolescents with mental disorders are more likely to have rich manifestations of NSSI (53, 54). In this study, the detection rate of NSSI among adolescents was 63.428%, higher than the results obtained by Huang et al. (55) in their investigation of 1,500 adolescent psychiatric outpatients (38.93%). In the study conducted by Seveke et al. (56), the detection rate among 130 hospitalized adolescent psychiatric patients (50.77%) was lower than what was found in this study. Why was the detection rate of NSSI so high in the present research? Studies suggests that emotional disorders are usually accompanied by NSSI behavior, and individuals with such emotional disorders are more likely to engage in it (55, 57). The percentage of adolescents with depressive disorders and NSSI was as high as 44% (58). For this reason, this study also considered the current presence of mental illness in the sample, finding that patients with depressive and bipolar disorders accounted for a significant proportion (58.85%); these two types of patients are susceptible to NSSI (59), which may have contributed to the high detection rate of NSSI in this study. More importantly, this work also illustrates an important issue: people with concurrent mental illnesses may be more likely to increase the detection rate of NSSI, which should be considered in the final analysis.

### 4.2. The predictive effect of adolescent NSSI on suicidal ideation

The results of this study show that there was a significant positive correlation between NSSI and suicidal ideation among the adolescent participants. In other words, NSSI significantly predicted adolescent suicidal ideation, and engaging in NSSI increased the risk of future suicidal thoughts and behaviors; these conclusions are consistent with previous studies (9, 60–62). This also validated Hypothesis 1. As stated in the introduction, although some studies have suggested that NSSI has an “anti-suicide” function for suicidal ideation, the most common reason for teenagers to adopt NSSI behaviors is to relieve their painful negative emotions (63). The self-injured person transfers or relieves their negative emotions through the NSSI behavior, which to some extent triggers physiological changes in the body (such as changes in endogenous opioid concentrations and the parasympathetic nervous system), thus causing the self-injured person to indulge in the transient pleasure provided by the NSSI behavior (64). However, research has found that as the frequency of individual NSSI acts increases, individuals become accustomed to the physical pain and fear caused by self-injury, and the probability of suicidal ideation consequently increases (65). This may also be the reason why the relationship between NSSI and suicidal ideation in the present study was not evident in the mild NSSI group, only in the moderate/severe NSSI group. From this, it can be seen that teenagers should be taught that NSSI behaviors represent a negative coping style and should be avoided (66). However, as the frequency of individual NSSI increases, the effectiveness and habituation of NSSI for pain relief may make such adolescents reluctant to abandon their NSSI behaviors. Because these feelings and thoughts are contradictory, individuals may regularly return to their NSSI behaviors. Moreover, rumination is a risk factor also related to suicidal ideation, leading to higher levels of suicidal ideation and risk while ruminating.

### 4.3. Mediating effect of rumination and decentering

This study found that rumination played a mediating role between NSSI and suicidal ideation. In other words, the higher the level of NSSI was, the higher the rumination level and resulting rate of suicidal ideation. This finding confirms previous results regarding the relationship between rumination and self-compassion in NSSI and suicidal ideation (67). Therefore, Hypothesis 2 was validated, which supports the DSM as the theoretical basis for explaining the relationship between NSSI and suicidal ideation in adolescents. Studies have shown that one of the motivations of NSSI is self-punishment (68–70) and people often express their disgust, anger, or feelings of degradation through self-injury. This is consistent with the CMT theory of rumination, which holds that rumination is usually caused by pathogenic beliefs of interpersonal guilt and that its unconscious goal is usually self-punishment (71). In order to achieve the purpose of self-punishment, the occurrence of self-injury may be accompanied by the emergence of rumination. Self-injured people who punish themselves usually carry with them the false belief that “it is of no meaning for me to live, and I am dragging others down.” Belittled and denied the value of their own lives, they believe they are a burden to others. This produces a sense of perceptual burden (72). The existence of perceptual burden can cause and intensify an individual’s suicidal ideation (73).

However, how does rumination affect suicidal ideation in adolescent NSSI? The DSM is not sufficient to explain this problem. This study found that decentering played a mediating role between rumination and suicidal ideation. In other words, in the NSSI group, the higher the level of rumination, the lower was the level of decentering and higher the level of suicidal ideation. Hypothesis 3 of the present study was therefore tested. As described in the introduction, self-injured persons experience more negative emotions and fewer positive emotions than do individuals with no NSSI behaviors (43), resulting in emotional dysregulation. As a core emotion regulation strategy, decentering was found to be inversely proportional to emotional dysregulation and could participate in individual cognitive reconstruction (74). Individuals suffering from NSSI because of emotional dysregulation (75) regulated their negative emotions by implementing self-injurious behaviors (76). The level of emotional dysregulation affected the occurrence of NSSI and amount of decentering and prompted individuals to increase self-injury and decrease decentering (when under certain conditions). In addition to self-injury, emotional dysregulation could also trigger substance abuse and other addictive behaviors (77), and eventually lead to suicidal ideation and other deteriorating symptoms (78).

In addition, through the examination of the chain mediation model, this study found that adolescent NSSI affected suicidal ideation through the chain mediation of rumination and decentering. Adolescents with high levels of NSSI also had high levels of rumination, leading to lower levels of decentering and higher levels of suicidal ideation; thus, Hypothesis 4 was verified. Furthermore, previous research has found that rumination was significantly negatively associated with decentering (41). Rumination is a self-centered, repetitive mode of thinking that focuses attention on negative stimuli, as opposed to decentering, which emphasizes an objective third-party perspective (79). Therefore, the level of rumination in self-injured individuals increases with the implementation of NSSI, while the intensification of rumination weakens the level of decentering, further increasing suicidal ideation and prompting individuals to cope with or escape negative life situations through suicide.

It should be added that this study found that NSSI also directly increased the degree of suicidal ideation by reducing the level of decentering; that is, decentering played a mediating role between NSSI and suicidal ideation, verifying Hypothesis 5. Previous studies have found that there is a significant positive correlation between NSSI and dissociative symptoms (30). NSSI individuals may rid themselves of the emptiness, numbness, and self-alienation of a dissociative state by increasing their suicidal ideation or even engaging in suicidal behavior. The findings of this study provide further theoretical explanations for the above results, showing that NSSI can negatively predict decentering, thus supporting PP theory (45, 46). The latter indicates that NSSI individuals may have a special cognitive model of self and the world, and the external environment results in biased NSSI behavioral responses such as biting. This biased response automatically stimulates the individual’s negative self-perception, and the level of decentering in their self-perception is thus reduced. This study also found that NSSI can directly affect suicidal ideation through the mediation of decentering. Specifically, NSSI behavior itself may lead to more dissociative symptoms related to individual automation, reducing the level of decentering and leading to an increase in the level of suicidal ideation.

In sum, this study found that NSSI can affect suicidal ideation through the mediation of rumination, which supports the theoretical view of the DSM and indicates that the relationship between NSSI and suicidal ideation could further be explained by use of this model in the future. On this basis, this study also found that NSSI can not only affect suicidal ideation through the chain mediation of the two variables of rumination and decentering, it also directly affects suicidal ideation though the mediation of decentering. Obviously, the two mediation paths of NSSI on suicidal ideation need to operate through decentering, as it plays a key role in the relationship between NSSI and suicidal ideation. It also shows that the introduction of the concept of decentering in this study expands the DSM model to explain the relationship between NSSI and suicidal ideation.

### 4.4. Application value, limitations, and future research directions

This study explored the relationship and mechanism operating between NSSI and suicidal ideation. We found that rumination and decentering played mediating roles in the influence of NSSI on suicidal ideation. Moreover, decentering can also play a mediating role between NSSI and suicidal ideation, indicating that decentering should play a key role between NSSI and suicidal ideation. These results provide references for NSSI behavior and suicide intervention.

In clinical interventions, cognitive therapy that encourages clients to challenge negative thinking and mindfulness therapy that has been proven to effectively alleviate the impact of rumination (80) and enhance individual decentering (81) should be used to address existing rumination phenomena and make individuals more aware of their irrationality, increase their problem-solving abilities, and help them to escape the negative cycle. Doing so will effectively reduce rumination (82), improve individual emotional regulation, and guide individuals to adopt positive coping styles and increase their adaptability. In addition, as a new intervention method, working memory training would help individuals reduce the difficulty of removing negative information, and could also be used in rumination interventions related to self-injury (83).

There were some limitations to this study. First, due to the difficulty of sampling adolescent NSSI participants, this study mainly adopted a cross-sectional survey design. However, this restricted our ability to make accurate causal inferences about the relationships among variables. In the future, it will be necessary to test the causal relationships through a longitudinal design. Second, the difficulty in sampling also led to an inability to balance the demographics, such as with regards to family residence and whether the participant was an only child. Finally, the samples for this study were sourced from a clinical population. In the future, sampling from the community as much as possible would further improve the external validity of the research results.

## 5. Conclusion

NSSI was found to be positively associated with suicidal ideation and rumination but negatively associated with decentering. Moreover, suicidal ideation was positively associated with rumination and negatively associated with decentering. Rumination was negatively associated with decentering. Additionally, NSSI positively predicted adolescents’ suicidal ideation. Rumination and decentering played a chain mediation role between NSSI and suicidal ideation. NSSI indirectly affected suicidal ideation along three paths: the independent mediating role of rumination, independent mediating role of decentering, and chain-mediating role of rumination and decentering.

## Data availability statement

The original contributions presented in the study are included in the article/supplementary material, further inquiries can be directed to the corresponding authors.

## Ethics statement

The studies involving human participants were reviewed and approved by the Biomedical Research Ethics Committee of Fujian Medical University. Written informed consent to participate in this study was provided by the participants’ legal guardian/next of kin.

## Author contributions

FH, ML, and QJ conceived the idea of the study. QJ and YZ collected the data. YZ and FH engaged in the analysis and interpretation of the data. QJ, YZ, and FH wrote the manuscript and revised the manuscript. FH is the first corresponding author. All authors designed the research, reviewed the manuscript, read, and approved the final version of the manuscript.

## Funding

This work was supported by the Natural Science Foundation of Fujian Province (2021J01816) and the School of Health at Fujian Medical University. The funders had no role in the study design, data collection and analysis, decision to publish, or preparation of the manuscript.

## Conflict of interest

The authors declare that the research was conducted in the absence of any commercial or financial relationships that could be construed as a potential conflict of interest.

## Publisher’s note

All claims expressed in this article are solely those of the authors and do not necessarily represent those of their affiliated organizations, or those of the publisher, the editors and the reviewers. Any product that may be evaluated in this article, or claim that may be made by its manufacturer, is not guaranteed or endorsed by the publisher.
